# Phoretic mite communities associated with *Ips typographus* and *Ips duplicatus* (Coleoptera: Scolytinae) in a Norway Spruce stand

**DOI:** 10.1007/s10493-025-01053-3

**Published:** 2025-08-25

**Authors:** Dragoș Toma, Gabriela Isaia, Minodora Manu, Dieter Carol Simon

**Affiliations:** 1 National Institute for Research and Development in Forestry “Marin Dracea”, Eroilor, 128, 077190 Voluntari, Romania; 2 Faculty of Silviculture and Forest Engineering, Transilvania University of Brașov, Șirul Beethoven 1, Brașov, 500123 România; 3 Department of Taxonomy, Ecology and Nature Conservation, Research Station Posada, Institute of Biology Bucharest of Romanian Academy, Splaiul Independenței, no. 296, Bucharest, Romania

**Keywords:** Bark beetle, Phoresy, Phoretic mites, *Picea abies*, Community, Romania

## Abstract

European spruce bark beetle *Ips typographus* (Linnaeus 1758) (Scolytinae) is considered the most destructive and aggressive pest of Norway spruce in Europe. Recently, *Ips duplicatus* (Sahlberg 1836) (Scolytinae), another species of bark beetle, that also affects *Picea* trees, has expanded its range westwards in Europe. In spruce stands, bark beetle populations are closely associated with various organisms such as fungi, nematodes, and mites. While phoretic mites and their relationship with *I. typographus* have been extensively studied in Europe, only single study has focused on the populations of phoretic mites associated with *I. duplicatus*. The aim of this study is to analyze and document the communities of phoretic mites and their complex relationships with these two bark beetles species. The research was conducted in a stand located at the lower limit of spruce, where the two pest species have developed outbreaks together. Over 50,000 beetles were collected using wing-type pheromone traps, of which 4,348 were analyzed for the determination of phoretic mites (2,413 *I. typographus*; 1,935 *I. duplicatus*). In total, nine species of phoretic mites were identified, of which only six were found on *Ips duplicatus*. Among the nine species, *Dendrolaelaps disetus* Hirschmann, 1960 (Digamasellidae), *Elattoma* sp. (Pygmephoridae), and *Paraleius leontonychus* Berlese, 1910 (Oribatulidae) are reported for the first time in Romania. The results showed that although *Ips typographus* beetles carried significantly more phoretic mites than *Ips duplicatus* throughout the entire flight period, both species exhibited similar peaks in phoresy rate. ONE-WAY PERMANOVA test revealed significant differences between the two phoretic mite communities, differences also highlighted by diversity indices. These differences are most likely due to the presence of certain mite species only on *I. typographus* beetles, as well as differences between the populations of common species. The phoretic attachment location on host beetles varied across mite and host beetle species.

## Introduction

Norway spruce (*Picea abies* (L.) H. Karst) is considered the most important gymnosperm species in Europe (Caudullo et al. 2016; Skrøppa 2003; Westin and Haapanen 2013), covering an area of approximately 30 million hectares (Jansson et al. 2013). In Romania, it is the second most widespread tree species (Sofletea and Curtu 2008), where, both in pure and mixed forests, it occupies around 28% of the country’s forested area (Sidor et al. 2015). Considering its ecological and especially economic importance, due to the exceptional quality of its wood (Caudullo et al. 2016; Westin and Haapanen 2013), the range of Norway spruce has been expanded over the past two centuries through artificial plantations outside its natural ranges (Caudullo et al. 2016; Jansson et al. 2013; Neţoiu et al. 2008), in areas characteristic of deciduous species (Jansson et al. 2013; Klimo et al. 2000).

Abiotic factors such as severe storms or extreme drought weaken and physiologically weeken Norway spruce stands, particularly those outside their natural range (Caudullo et al. 2016; Spiecker 2000), thus creating favorable conditions for the outbreaks of bark beetles (Caudullo et al. 2016; Simionescu et al. 2000; Wermelinger 2004). The most destructive and aggressive pest among the bark beetles of Norway spruce is the European spruce bark beetle, *Ips typographus* (Linnaeus 1758) (Caudullo et al. 2016; Marini et al. 2013; Netherer et al. 2019; Simionescu et al. 2000; Wermelinger 2004). Considered to be a species that normally colonizes dying trees, during mass outbreaks it can also attack healthy trees (Simionescu et al. 2000; Wermelinger 2004; Weslien et al. 1989). In the past century, *I. typographus* outbreaks have led to the partial or total dieback of millions of cubic meters of spruce trees in Europe (Grégoire and Evans 2004).

Recently, *Ips duplicatus* (Sahlberg 1836), another bark beetle species of conifers, has expanded its range into the countries of Central and Southeastern Europe. (Holusa et al. 2010; Olenici et al. 2009, 2022; Wermelinger et al. 2020), being considered an invasive species in Europe (Olenici et al. 2022; Zúbrik et al. 2006) and added to quarantine lists by the European Union (EPPO 2025). Although it attacks several species within the genus *Picea* and occasionally *Pinus*, *Larix*, and *Pseudotsuga* (Duduman et al. 2013; Holuša and Grodzki 2008; Kašák and Foit 2015; Pfeffer and Knížek 1995; Wermelinger et al. 2020), it prefers Norway spruce stands, particularly those outside their natural range (Olenici et al. 2009, 2022; Wermelinger et al. 2020), where it causes outbreaks of varying intensity (Grodzki 2003; Holuša et al. 2003; Olenici et al. 2009, 2011). Sometimes, the attack of *I. duplicatus* on Norway spruce trees occurs together with *I. typographus* (Grodzki 2012), as the species share similar behavior and biology (Wermelinger et al. 2020). In Romania, *Ips duplicatus* is now present in most Norway spruce cultivation areas (Olenici et al. 20022).

Tree dieback in outbreak areas is not only the result of bark beetle attacks but also of pathogenic fungi with which the beetles associate and introduce into the wood (Lieutier 2002; Linnakoski et al. 2016; Moser et al. 2010; Paine et al. 1997; Wermelinger 2004). *I. typographus* is considered the bark beetle species most associated with pathogenic fungi (Krokene and Solheim 1996). Numerous studies have shown that, in addition to pathogenic fungi, bark beetle populations are closely linked to other organisms such as nematodes or mites (Forsse 1987; Hofstetter et al. 2015; Moser and Bogenschütz 1984). Phoretic mite species use bark beetles for dispersal through a phenomenon called phoresy (Bartlow and Agosta 2021; Camerik 2010; White et al. 2017). For dispersal to occur, phoresy must include three fundamental stages: host location, attachment to the host, and detachment at the appropriate time (Bartlow and Agosta 2021). Phoresy does not involve parasitic relationships, although it can become antagonistic to host species over time. Many phoretic organisms attached to the host body can somewhat affect the host’s locomotion ability (Gwiazdowicz et al. 2011). Moreover, when a phoretic relationship forms between two organisms, it can evolve into a parasitic relationship (White et al. 2017).

In the case of *I. typographus*, following the significant outbreaks it caused in Norway spruce stands across Europe (Bakke 1983; Simionescu et al. 2000; Wermelinger 2004), interest in this pest has grown considerably, leading to intensive studies on the relationship between bark beetle populations and their phoretic mites. To date, numerous studies have focused on this aspect, with over 60 species of phoretic mites being identified in close association with the European spruce bark beetle, *I. typographus* (Gwiazdowicz 2008; Skorupski and Gwiazdowicz 1998). On the other hand, the phoretic mite species, their abundance, and the relationship they have with the bark beetle *I. duplicatus* have been very little studied, with only one study conducted in Europe (Čejka and Holuša 2014). Furthermore, a comparative analysis of the phoretic mite populations associated with the two bark beetle species, which coexist and together attack trees in an outbreak, has not been conducted.

In this context, through this study, we aim to determine and analyze the following aspects: (i) the temporal dynamics of phoresy during the flight activity of both bark beetle species; (ii) characterize the diversity and population dynamics of phoretic mites associated with *I. typographus* and *I. duplicatus*; (iii) the attachment preference of phoretic mite species based on their host.

## Materials and methods

### Study area

**Fig. 1:**
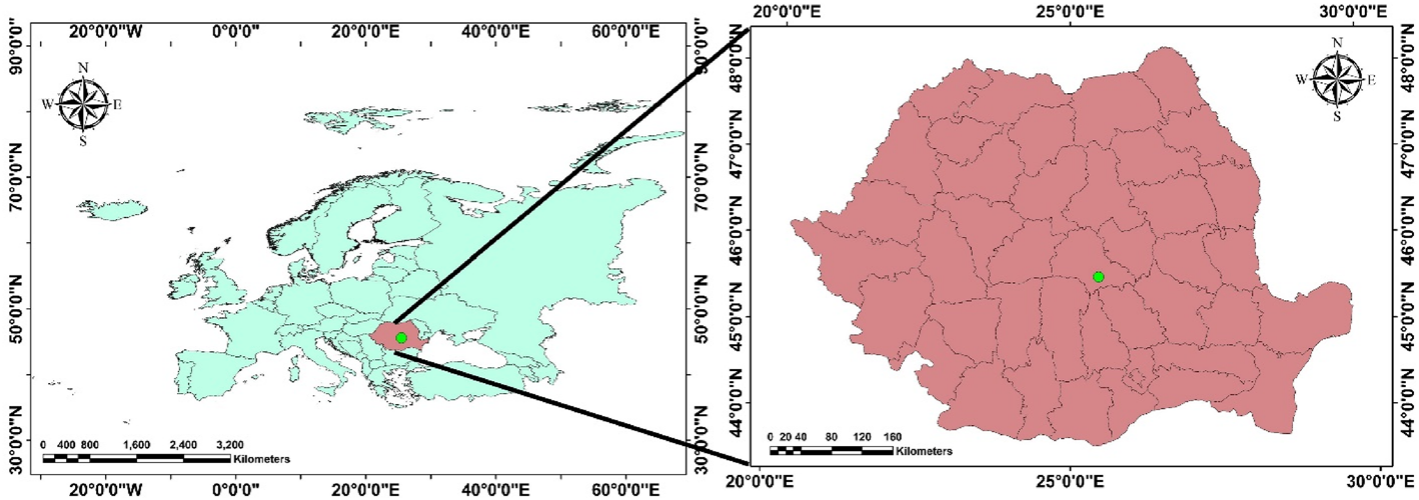
Location of the study area (Esri, Maxar, GeoEye, Earthstar Geographics, CNES/Airbus DS, USDA, USGS, Aero Grid, IGN, and theGIS User Community)

The research was conducted in a Norway spruce stand near Râșnov (45°35 N; 25°28 E), Brașov County, managed by the Râșnov Town Forest Administration, in forest compartment 72B (Fig. 1; Table 1). In this region, *Ips duplicatus* has been present since 2011 (Duduman et al. 2011) and, in association with *Ips typographus*, has contributed to infestations of standing trees.

**Table 1:** Forest stand characteristics

Management unit	Area (ha)	Habitat type^a^	Altitude (m)	Exposition	Composition %	Age (years)	Canopy cover
72B	2.5	South-Eastern Carpathian forests with *P. abies*,* Fagus sylvatica*,* Abies alba and Pulmonaria rubra*	715	NV	100 *P. abies*	80	0.8

^a^According to Doniţă et al. (2005)

### Collection and analysis of entomological material

Bark beetles were collected using six intercept traps (wing type), three baited with a commercial pheromone specific to *I. typographus* and three baited with a commercial pheromone specific to *I. duplicatus*, produced by the Raluca Ripan Institute of Chemistry, Romania (Table 2).

**Table 2:** The pheromone types used and their compositon

Bark beetles species	Pheromone type	Pheromone composition
*Ips typographus*	AtraTYP	2-methyl-3-buten-2-ol (-)-cis-verbenol
*Ips duplicatus*	AtraDUP	E-myrcenol, Ipsdienol, 2-methyl-3buten-2-ol

The distance between the two trap arrays was 25 m, while the distance between traps within the arrays was 50 m. The traps were set up on May 2, 2023, at 10–12 m from the forest edge, and bark beetles were collected every 7–11 days throughout the entire growing season, with the last collection in mid-September.

The collected beetles were stored in a freezer at a temperature of −5 °C to prevent the detachment of phoretic mites from their hosts (Moser and Bogenschuütz 1984; Paraschiv and Isaia 2020). Subsequently, from each capture, the bark beetles were identified, and a random sample of 50 specimens was retained for the analysis of phoretic mites. If the number of bark beetles per trap was less than 50 specimens, all available specimens were analyzed (Paraschiv and Isaia 2020). Insect sex determination was performed through dissection based on their genitalia (Duduman 2019; Duduman et al. 2022). Regarding the phoretic mites on the bodies of the beetles, after the species identification their numbers were recorded based on the location of attachment to the beetle bodies. After beetles were identified to species, the hosts were examined for mites using Zeiss stereo microscope. The attachement location of the mites was determined by subdividing the host body into several parts: head, thorax, abdomen, first, second and third pair of legs, elytral declivity, and under elytra (Paraschiv and Isaia 2020). Mites were stored in 96% ethanol in a −20 °C freezer for later slide mounting. The mite species were slide mounted in polyvinyl alcohol–lactic acid mixture (PVA) mounting medium, and cured on a slide warmer at 45 °C for 3–4 days (Krantz and Walter 2009). Slide mounted mites were identified examined using a Zeiss Axio Scope A 1 compound microscope, and identified to species using identification keys provided byfrom the scientific literature (Ghiliarov and Bregetova 1977; Kinn 1968; Khaustov 2000; Moser and Bogenschuütz 1984; Rahiminejad et al. 2011; Trach and Khustov 2018).Voucher specimens of all mite species detected in this study are stored at the laboratory of the “Marin Drăcea” National Institute for Research and Development in Forestry, Voluntari, Ilfov.

### Statistical analysis

The zoocenological analysis of mite communities was evaluated using the dominance index (D), categorized as follows: eudominant (> 30%), dominant (15.1–30%), sub-dominant (7.1–15%), resident (3.1–7%), and sub-resident (< 3%); and frequency (F) with the following classes: euconstant (> 50%), constant (30.1–50%), subconstant (15.1–30%), accessory species (5.1–15%), and accidental occurrence (< 5%), as used in other studies (Gwiazdowicz et al. 2011; Paraschiv and Isaia 2020). Dominance (D) was calculated by dividing the total number of individuals of a phoretic mite species by the total number of phoretic mites. Frequency (F) was determined as the ratio of the total number of bark beetle with a specific species of phoretic mites to the total number of analyzed beetles. Phoresy rate was determind by the ratio between beetles that carried mites and total number of beetles analyzed.

The application of the Shapiro-Wilk test confirmed the normal distribution of the data, and Levene’s test verified the homogeneity of the data, thus meeting the requirements for the application of parametric tests. In order to evaluate if the intensity of phoresy and the phoresy rate were influenced by factors such as bark beetle species, collection date or beetle sex, and to determine the body parts most frequently occupied by phoretic mites on each bark beetle species and the attachment preference of each mite species for a specific body part of their hosts, One-Way ANOVA analysis of variance was conducted. The significance level of the differences between variables was established using Tukey’s multiple test. Due to the low number of attached phoretic mites the attachment on the first, second and third pair of legs and the head in the case of *I. typographus* and the attaachment on the head and the third pair of legs in the case of *I. duplicatus* were not included in the statistical analyses. If the number of specimens for a species was insufficient for this analysis, or if individuals of a species did not exhibit a specific preference for any particular body part, it was noted that the species had no distinct attachment preference.

The characterization and evaluation of the differences between the phoretic mite populations of the two bark beetle species were determined using diversity indices such as the Shannon diversity index (H’), Simpson index (1-D), Evenness index (e^H/S), and Berger-Parker index, along with the PERMANOVA test (Anderson 2014; Isaia et al. 2022; Magurran 2004). The application of the PERMANOVA test on the two communities was conducted based on Bray-Curtis dissimilarity and 9999 random permutations. To ensure that the effect of the dominant species (*Dendrolaelaps quadrisetus* Berlese, 1920) did not overly influence the analysis results, the data were transformed using the log10(x + 1) function (Isaia et al. 2022). To visualize the differences between the phoretic mite assemblage populations of the two bark beetle species, non-metric multidimensional scaling (NMDS) was employed (Revainera et al. 2019).

For the primary data processing and graphical presentation of the dynamics of the phoretic rate in relation to the capture levels of the two bark beetle species, Microsoft Excel (Microsoft Corp., Redmond, Washington, USA) was used. Normality testing, homogeneity, and statistical differences were performed using STATISTICA 8.0 software (Weiß 2007). The diversity indices, PERMANOVA test, and NMDS were conducted with PAST 4.03 (Hammer and Harper 2001).

## Results

### The dynamics of insect flight and phoresy rates

**Fig. 2:**
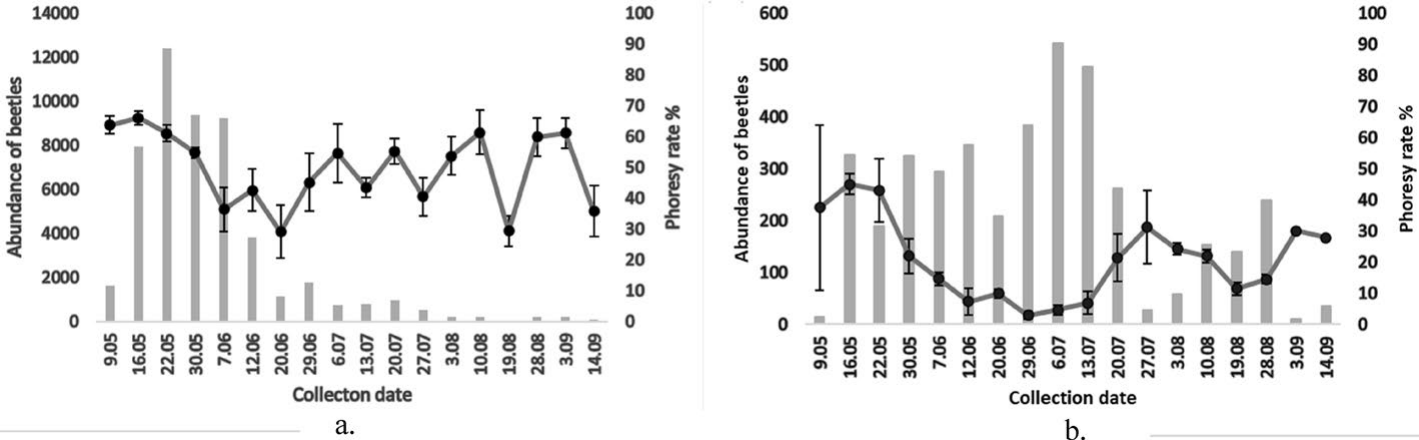
Phoresy rate (±SE) (line) and flight activity of *Ips typographus* (**a**) and *Ips duplicatus* (**b**) (columns) based on pheromone trap captures in 2022

During the entire vegetation season, a total of 55,276 bark beetles were captured in the pheromone traps, 51,222 of them were *I. typographus* and 4,054 were *I. duplicatus*. The two species reached their peak flight intensity at different times. For *I. typographus*, the highest number of beetles was recorded in the second half of May, while for *I. duplicatus*, the highest number of beetles was recorded in the first half of July (Fig. 2).

Out of the total captured beetles, 2,413 specimens of *I. typographus* were subsequently analyzed to determine the phoretic mites, resulting in a phoresy rate of 49.3%. For *I. duplicatus*, 1,935 beetles were analyzed, of which only 20.6% carried phoretic mites on their bodies. The differences between the two species were significant (df = 1, f = 133.8, *p* < 0.05). Both *I. typographus* males (50.7) and *I. duplicatus* males (23.3%) carried more phoretic mites than females (47.9%; 17.8%), but there were no significant differences (df = 1, f = 0.7, *p* > 0.05; df = 1, f = 2.1, *p* > 0.05).

Regarding the phoresy rates over time, significant fluctuations were observed for both *I. typographus* (df = 17, f = 5.2, *p* < 0.05) and *I. duplicatus* (df = 17, f = 4.8, *p* < 0.05). For *I. typographus* (Fig. 2a), the phoresy rate reached its first peak at the beginning of the flight (May 9– May 16), followed by two more peaks at the beginning of August (August 3– August 10) and at the end of the flight (August 28– September 3). Although at a considerably lower intensity, the phoresy rate for *I. duplicatus* exhibited peaks like those recorded for *I. typographus* (Fig. 2b). The first and most significant peak *for I. duplicatus* was recorded at the beginning of the flight (May 9– May 16), followed by two additional peaks at the end of July (July 20– July 27) and at the end of the flight (August 28– September 3).

### Species composition and zoocenological analysis

The phoretic mites identified on the body of *I. typographus* belong to nine species, namely: *D. quadrisetus* (Mesostigmata: Digamasellidae), *Dendrolaelaps disetus* Hirschmann, 1960 (Mesostigmata: Digamasellidae), *Elattoma* sp. (Trombidiformes: Pygmephoridae), *Histiostoma piceae* Scheucher, 1957 (Astigmata: Histiostomatidae), *Paraleius leontonychus* Berlese, 1910 (Oribatida: Oribatulidae), *Pleuronectocelaeno austriaca* Vitzthum 1926 (Mesostigmata: Celaenopsidae), *Proctolaelaps fiseri* Samsinak, 1960 (Mesostigmata: Asciadae), *Trichouropoda polytricha* Vitzthum, 1923 (Mesostigmata: Trematuridae), and *Uroobovella ipidis* Vitzthum, 1923 (Mesostigmata: Urodinychidae) (Table 3). The most frequent and dominant species on the bodies of *I. typographus* beetles was *D. quadrisetus*, which represented more than half of the total (63.46%). Specimens of this species, along with specimens of the species *U. ipidis*, *H. piceae*, and *T. polytricha*, accounted for a total of 98% of the entire mite population, while the other species were classified as having accidental dominance and accidental occurrence frequency.

Only six species of mites were found on the bodies of *I. duplicatus* beetles, with their dominance being more evenly distributed compared to *I. typographus* (Table 3). In this case, the most abundant and dominant species was *Elattoma* sp., followed by *D. quadrisetus* and *T. polytricha*, together represented approximately 86% of the total phoretic mite population. The frequency of the mite species identified on *I. duplicatus* was much lower, not exceeding 10% for any of the species. The species *Elattoma* sp. was the only species that was more abundant on the *I. duplicatus* beetles compared to the *I. typographus* beetles.

**Table 3:** Phoretic mite species of *Ips typographus* and *Ips duplicatus*, their abundance, dominance, frequency and feeding behavior

Order	Family	Species	Feeding Behavior^a^	*Ips typographus*			*Ips duplicatus*		
				Abundance	Dominance (%)^b^	Frequency (%)^b^	Abundance	Dominance (%)^b^	Frequency (%)^b^
Mesostigmata	Digamasellidae	***Dendrolaelaps quadrisetus***	Predacious	1963	63.7	34.3	186	31.9	9.6
Mesostigmata	Digamasellidae	***Dendrolaelaps disetus***	Predacious	2	0.1	0.1	0	–	–
Trombidiformes	Pygmephoridae	***Elattoma*** **sp.**	Mycetophagous	5	0.2	0.2	197	33.7	5.7
Astigmata	Histiostomatidae	***Histiostoma piceae***	Unknown	376	12.2	8.2	62	10.6	2.3
Oribatida	Oribatulidae	***Paraleius leontonychus***	Unknown	11	0.3	0.5	0	–	–
Mesostigmata	Celaenopsidae	***Pleuronectocelaeno austriaca***	Predacious	9	0.3	0.3	0	–	–
Mesostigmata	Ascidae	***Proctolaelaps fiseri***	Predacious	27	0.9	0.9	1	0.2	0.1
Mesostigmata	Trematuridae	***Trichouropoda polytricha***	Unknown	298	9.6	9.2	121	20.7	4.6
Mesostigmata	Urodinychidae	***Uroobovella ipidis***	Unknown	391	12.7	9.3	17	2.9	0.8

^a^According to Hofstetter et al. (2014)

^b^Classification according to Gwiazdowicz et al. (2011)

### Community diversity and structure

The diversity indices show significant differences between the two analyzed communities of phoretic mites (Table 4). In the case of the Shannon (H), Simpson, and Evenness indices, the results indicate that the phoretic mite community associated with the bark beetle *I. duplicatus* is more diverse and uniformly distributed. The considerably higher value recorded for the Berger-Parker index in the phoretic mite community of *I. typographus* indicates that it was dominated by a single species.

**Table 4:** Diversity indices of the phoretic mites of two bark beetles

Diversity index	*Ips typographus*	*Ips duplicatus*
Shannon_H	1.13	1.409
Evenness_e^H/S	0.344	0.682
Simpson_1-D	0.5564	0.7297
Berger-Parker	0.6346	0.3373

**Fig. 3:**
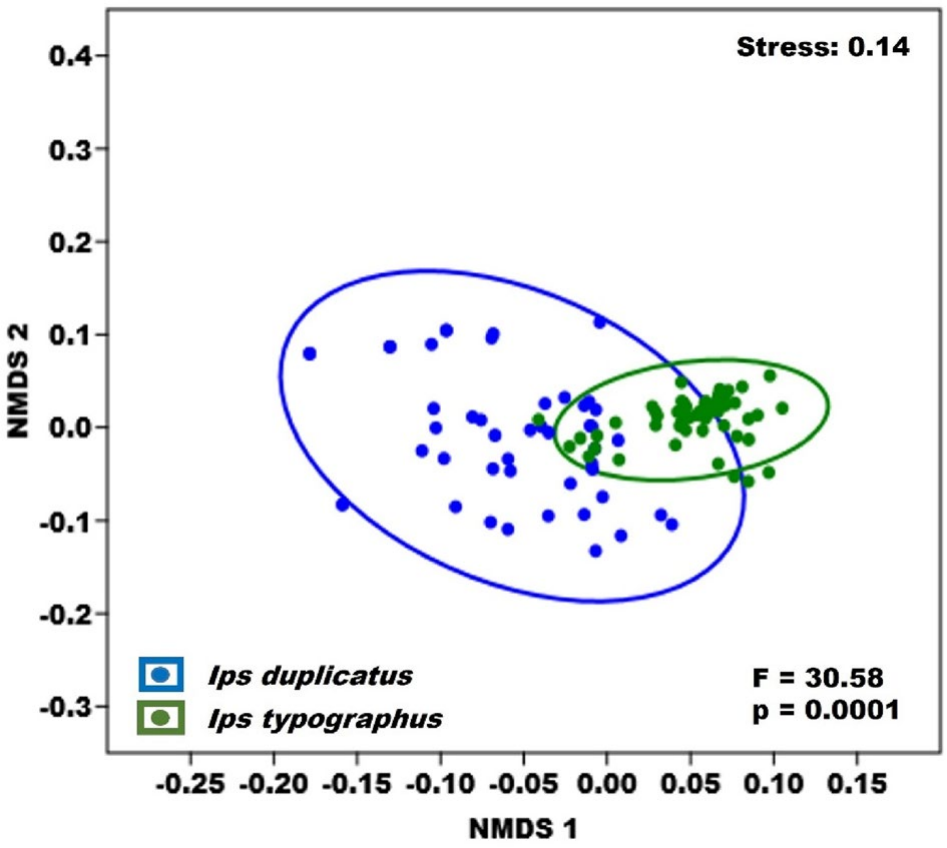
Non-metric, multidimensional scaling (NMDS) of the phoretic mites on *Ips typographus* and *Ips duplicatus* represented as Bray-Curtis dissimilarity. PERMANOVA test with 9999 random permutations was used for the comparison

The result of the permutational analysis of variance (PERMANOVA) indicates a significant difference in the composition of the phoretic mite communities associated with the two bark beetle species (F = 30.6 *p* < 0.001). The NMDS analysis shows that, although the two populations do not completely differentiate from each other, the community associated with the bark beetle *I. typographus* is much more centered, showing a tendency to separate from the community of *I. duplicatus* (Fig. 3).

### Location of phoretic mites on the bodies of bark beetles

**Fig. 4:**
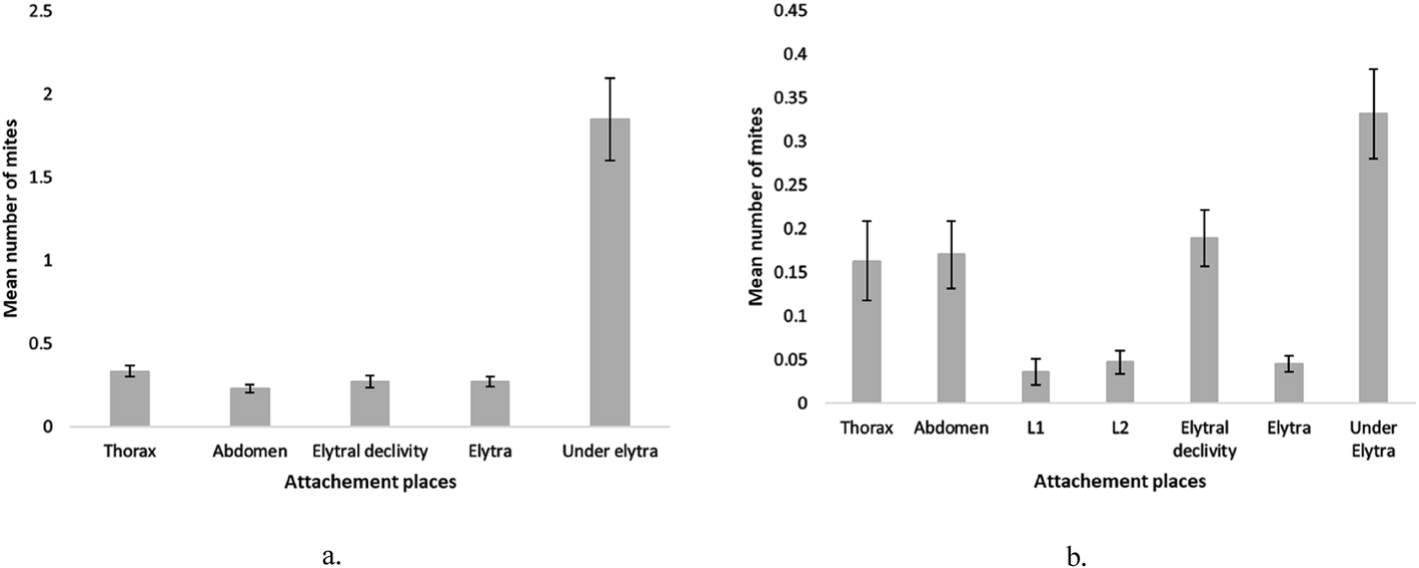
Mean number of phoretic mites (±SE) on the body of *Ips typographus* (**a**) and *Ips duplicatus* (**b**)

Significant differences were recorded between the average number of phoretic mites attached to different body parts for both bark beetle species. For *I. typographus*, the highest average number of mites was recorded under the elytra (1.9, significantly differing from the other parts (df = 4, f = 38.4, *p* < 0.05): thorax (0.3), elytral declivity (0.3), elytra (0.3), and abdomen (0.2) (Fig. 4a). In the case of *I. duplicatus*, the highest average number of mites was also found under the elytra (0.3), significantly differing from the other parts (df = 6, f = 10.4, *p* < 0.05) (Fig. 4b). The next preferred attachment sites were the elytral declivity (0.2), abdomen (0.2), and the thorax (0.2). Although the average number of phoretic mites differs between the two hosts, the results regarding the localization of mites on their bodies are similar, with most mites located under the elytra in both species, followed by the thorax, abdomen, pairs of legs, and elytra. Additionally, the less frequented areas for mites were the heads of the hosts and pairs of legs 3 in both species of bark beetles (Fig. 4).

The distribution of phoretic mites on the beetles varied according to both mite species and host beetle (Table 5). For both *I. duplicatus* and *I. typographus*, most specimens of *D. quadrisetus* were identified under the hosts’ elytra, with the differences statistically supported by the other locations (DF 8; F 52.3; *p* < 0.05; DF 8; F 44.9; *p* < 0.05). Although the few specimens of *Elattoma* sp. detected on *I. typographus* did not show a preference for any specific body part, those identified on *I. duplicatus* were mainly located in the area between pairs of legs 1 and 2, on the abdomen or thorax, significantly differing (DF 8; F 4.8; *p* < 0.05) from the rest of the body parts.

Notable differences in attachment between the two hosts were also observed for the species *H. piceae*, *T. polytricha*, and *U. ipidis*. However, it is worth noting a particular behavior in some specimens of *H. piceae* located under the hosts’ elytra in hyperphoresis with some specimens of *D. quadrisetus*. The only specimen of *P. fiseri* identified on *I. duplicatus* was found on the abdomen, whereas specimens identified on *I. typographus* did not show a specific preference, with most located under and on the elytra (DF 8; F 1.4; *p* > 0.05). The attachment preference of the species *P. austriaca* on *I. typographus* was predominantly under the elytra (DF 8; F 3.2; *p* < 0.05). *P. leontonychus* did not show a significant preference for the host body (DF 8; F 1; *p* > 0.05). The only two specimens of *D. disetus* were identified on the thorax of *I. typographus*.

**Table 5:** Main location of mites on the host’s body

Species	Distribution of mites on the body of *Ips duplicatus*		Distribution of mites on the body of *Ips typographus*	
	Location	Proportion of total mites (%)	Location	Proportion of total mites (%)
*Dendrolaelaps quadrisetus*	Under elytra	95	Under elytra	88
*Dendrolaelaps disetus*	-		Thorax	100
*Elattoma *sp*.*	Abdomen	34	No preference	
	Thorax	31		
*Histiostoma piceae*	Abdomen	29	Elytra	36
	Thorax	26	Abdomen	23
*Paraleius leontonychus*	-		No preference	
*Pleuronectocelaeno* *austriaca*	-		Under elytra	78
*Proctolaelaps fiseri*	Abdomen	100	No preference	
*Trichouropoda polytricha*	Elytral declivity	79	Elytral declivity	36
			Thorax	23
*Uroobovella ipidis*	Thorax	24	Thorax	34
	Abdomen	24	Pairs of legs 1	25

## Discussion

### The flight activity of bark beetles and the dynamics of phoresy

From its first report in Romania in the mid-20th century (Negru and Ceianu 1957) until now, the northern bark beetle *I. duplicatus* has become a commonly encountered species in Romania, particularly in spruce forests outside its natural range (Olenici et al. 2022). In the sample area where this study was conducted, this pest was reported for the first time in 2011 (Duduman et al. 2011). Several studies indicate that *I. duplicatus* is a secondary pest that primarily colonizes trees aged between 30 and 70 years in the upper part of the crown, where the bark is thinner (Bakke 1975; Lekander et al. 1977; Postner 1974), and typically occurs in association with other bark beetles (Grodzki 2012; Duduman et al. 2013). The secondary character of this pest is also reflected in our study’s results, where captures recorded for *I. typographus* are significantly higher than those for *I. duplicatus*. The advanced age of the trees in the sample area does not suit the preferences of *I. duplicatus*, thereby maintaining the population at a low level.

The fact that *I. typographus* was the dominant species may also explain the preference of phoretic mites in choosing hosts for transportation to other habitats. The rate of phoresy for *I. typographus* beetles varied between 29% and 65%, with an average of 49.3%, representing the highest phoresy rate recorded in Romania (Manu et al. 2017; Paraschiv and Isaia 2020; Poliță et al. 2016) and in Europe (Gwiazdowicz et al. 2011, 2012; Zach et al. 2016; Milosavljević et al. 2022; Moser and Bogenschuütz 1984; Moser et al. 1989a; Takov et al. 2009). The differences between previous studies and the current findings can be attributed to several possible factors, starting from the method of preserving the bark beetles up to the moment of their analysis. In the case of beetles preserved in alcohol (ethanol), there is a risk that some phoretic mites may detach (Moser and Bogenschuütz 1984), which is the main reason why the beetles collected during the study were stored as quickly as possible at negative temperatures in freezers. Additionally, other factors that could influence the capture rate of phoretic mites might include the phase of the bark beetle outbreak (Vázquez and Haydeé 2018), the dynamics and density of the host beetle population (Paraschiv and Isaia 2020), or the location where the beetles overwinter, with those overwintering in the litter having lower chances of acting as vectors in the dispersal of phoretic mites (Annila 1969). These hypotheses may explain why *I. duplicatus* beetles carried significantly fewer phoretic mites than *Ips typographus*. The phoresy rate of *I. duplicatus* beetles varied from 3 to 45%, with an average of 20.6%. The fact that adult *I. duplicatus* primarily overwinter in the litter (Olenici et al. 2009; Onyśko and Starzyk 2011; Zhang 1995) may explain the low capture percentage of phoretic mites; however, from the results obtained (Fig. 1b), the highest percentages were reached at the beginning of the flight of the hibernating generation, although they did not reach the values of the hibernating generation of *I. typographus* (Fig. 1a). The hypotheses suggesting that outbreak phase, density, and population dynamics may influence the relationship between phoretic mites and bark beetles appear to be closer to the truth.

Given that *I. duplicatus* is a new species in the Romanian fauna and its presence in the study area was reported only in 2011, this may explain the low capture percentage. Furthermore, some species of mites might prefer individuals of the dominant species in the outbreak over other secondary species. In this context, the research conducted by Poliță et al. (2016), which analyzed the relationship between phoretic mites and bark beetles *I. typographus* and *Pityogenes chalcographus* (Linnaeus 1761) in spruce stands aged 80–110 years, yielded similar results, with *I. typographus* specimens transported a higher number of mites than *P. chalcographus*. Additionally, another study analyzing the populations of phoretic mites associated with bark beetles of pine trees in Portugal showed that the specimens of the primary pest *Ips sexdentatus* (Boerner 1776) carried more mites than the specimens of the secondary pest *Hylurgus ligniperda* (Fabricius 1787) (Vissa et al. 2019).

The bark beetle species analyzed in this study produce two generations per year (Olenici et al. 2009; Simionescu et al. 2000). For both species, the highest phoretic mite loads was recorded during the initial phase of flight activity, which coincides with the emergence of the overwintering generation. Subsequently, the phoretic rate in both bark beetle species reached a lower peak during the flight activity period of filial generation. These findings suggest that the optimal dispersal period for phoretic mites coincides with the flight activity of both the overwintering and filial generations, which aligns with results reported in previous studies (Čejka and Holuša 2014; Holuša and Čejka 2020; Paraschiv et al. 2018; Paraschiv and Isaia 2020). Phoretic mites synchronize their development with that of bark beetles to exploit the most favorable moment for dispersal (Paraschiv and Isaia 2020; Bajerlein et al. 2024). Thus, when overwintering beetles begin to fly, the mites are already prepared to attach to their bodies and be transported to a new host tree. Furthermore, Holuša and Čejka (2020) suggest that the time required for *D. quadrisetus* to develop back into the deutonymphal stage corresponds with the beginning of the filial generation’s flight activity. This may also be the case for other mite species that disperse exclusively in the deutonymphal stage, such as Uropodina species (Bajerlein et al. 2024). This may explain the high number of phoretic mites observed during the filial generation in this study. Although phoretic mites did not show a preference for the sex of the beetles from either species, a finding confirmed in other studies (Paraschiv et al. 2018; Paraschiv and Isaia 2020), males transported more mites than females. A similar behavior was also observed in the phoretic mites associated with beetles of the genera *Oryctes arabicus* and *Nicrophorus investigator* (Al-Deeb et al. 2012; Grossman and Smith 2008). Both studies suggest that certain mite species can distinguish the sex of the host beetle based on emitted pheromones and preferentially attach to males, likely due to their larger body size. Although size-based preference for males is unlikely in *I. typographus* and *I. duplicatus*, an alternative hypothesis is that mites preferentially attach to males because they are the first to initiate flight and spend more time in the air, increasing the chances of dispersal (Wermelinger 2004).

### Species composition and zoocenological analysis

The number of phoretic mite species identified in this study associated with *I. typographus* represents the highest recorded in Romania to date (Paraschiv and Isaia 2020; Poliță et al. 2016; Manu et al. 2017), and is comparable to findings from several European studies (Burjanadze et al. 2008; Holuša and Čejka 2020; Milosavljević et al. 2022; Takov et al. 2009), though it remains considerably lower than the number of phoretic mites identified in Germany (Moser and Bogenschuütz 1984), Sweden (Moser et al. 1989a), Poland (Gwiazdowicz et al. 2012, 2015), and Finland (Penttinen et al. 2013). The differences between these results can be explained by the methods of preserving and storing entomological material (Paraschiv and Isaia 2020), the specifics of the area where the insects were collected, the total number of beetles analyzed (Gwiazdowicz et al. 2011), or the methods used for identifying mites, whether on the bodies of the insects or in their galleries. For example, a study conducted in Russia that analyzed mites associated with *I. typographus* both on their bodies and from galleries identified over 60 species of phoretic mites closely linked to their hosts (Khaustov et al. 2018).

The only study that focused on identifying species of mites associated with *I. duplicatus*, in Europe, identified only 3 species of phoretic mites on the bodies of the insects (Čejka and Holuša 2014), a significantly lower number compared to the number of species identified in this study. However, it is worth mentioning that the study in the Czech Republic did not target *I. duplicatus* beetles throughout the entire vegetation season but only a small sample of insects collected at the beginning of the flight period. Of the six identified species, *H. piceae* and *P. fiseri* are recorded for the first time as forming phoretic associations with this host, aspect that can be explained by the large number of hosts used by these two species for transportation to other habitats (Hofstetter et al. 2015; Khaustov et al. 2018).

All identified species are relatively common and have been reported in previous studies. The most abundant and dominant species, *D. quadrisetus*, is one of the most frequently encountered phoretic mite species in the Northern Hemisphere, having been recorded in association with more than 25 bark beetle species (Khaustov et al. 2018; Knee et al. 2013). This species exhibits a generalist behavior regarding its phoretic host and inhabits a wide range of habitats (Moser 1996). The fact that this species was the most frequently encountered is not unexpected, as similar results have been reported in other studies (Gwiazdowicz et al. 2011, 2015; Poliță et al. 2016; Manu et al. 2017; Paraschiv and Isaia 2020; Holuša and Čejka 2020). On the contrary, the other species from the genus *Dendrolaelaps* identified in this study, *D. disetus*, is specific to *I. typographus* (Hofstetter et al. 2015) and has so far been found in Germany (Moser and Bogenschuütz 1984) and Poland (Skorupski and Gwiazdowicz 1998). Species from the genus *Dendrolaelaps* are predators, typically feeding on small organisms found in the galleries of bark beetles (Kinn 1983). However, several studies indicate that *D. quadrisetus* may also increase the mortality of bark beetles (Penttinen et al. 2013) by consuming their eggs and larvae (Maslov 2006; Pernek et al. 2008; Khaustov et al. 2018). Similar feeding behavior is exhibited by *P. fiseri* and *P. austriaca*. Like *D. quadrisetus*, *P. fiseri* is a generalist phoretic mite species, reported on several species of bark beetles (Hofstetter et al. 2015; Khaustov et al. 2018) in various habitats across Eurasia and North America (Khaustov et al. 2018). The low number of specimens identified in this study aligns with results from other studies (Penttinen et al. 2013; Paraschiv and Isaia 2020; Poliță et al. 2016), often being classified as a rare, accidental species. *P. austriaca* has so far been found in association with *I. typographus* (Hofstetter et al. 2015), *Scolytus scolytus* (Moser et al. 2010), and *Scolytus laevis* (Vitzthum 1926), having been reported in Romania (Manu et al. 2017), Poland (Gwiazdowicz et al. 2015), the Czech Republic (Holuša and Čejka 2020), and Austria (Moser et al. 2010; Vitzthum 1926). These reports may indicate that the range of this species is in Central and Eastern Europe. The low number of specimens may be attributed to the size of this phoretic mite species, which may reduce the probability of successful attachment to the host (Moser et al. 1989a).

The two species from the infraorder Uropodina, namely *T. polytricha* and *U. ipidis*, are commonly found in high abundance in several studies focusing on the phoretic mites associated with the bark beetle *I. typographus* (Moser et al. 1989a); Takov et al. 2009; Penttinen et al. 2013; Manu et al. 2017; Khaustov et al. 2018; Paraschiv and Isaia 2020; Holuša and Čejka 2020) and *I. duplicatus* (Čejka and Holuša 2014), as well as other species of bark beetles (Hofstetter et al. 2015). The nature of the relationship between these two mite species and their hosts remains unclear; however, they most likely utilize the insects exclusively as a means of transport (phoresy) (Paraschiv and Isaia 2020). However, some studies suggest that species from the genus *Trichouropoda* and *Uroobovella* may act as vectors for spores of fungi that alter wood color (Cardoza et al. 2008; Roets et al. 2014)d *polytricha* could be a predator of nematodes in the galleries of bark beetles (Kinn 1982).

*H. piceae* inhabits the galleries of many bark beetles (Pernek et al. 2008, 2012; Hofstetter et al. 2015; Wirth et al. 2016) across Eurasia, showing a greater affinity for habitats rich in fungal spores (Hofstetter et al. 2013). Specimens of this species can be vectors for certain pathogenic fungi that significantly reduce the resistance of host trees, ultimately leading to their death (Moser et al. 1989b).

Although *P. leontonychus* is most often found in low abundance (Moser and Bogenschuütz 1984; Pernek et al. 2008; Cilbircioğlu et al. 2021), it is a species with a wide distribution in the bark galleries of many insects (Ahadiyat and Akrami 2015). Its feeding behavior is unknown, although some authors suggest that it may be a detritivorous species (Pernek et al. 2008; Penttinen et al. 2013). Additionally, this species may act as a vector for pathogenic fungi (Moser et al. 1989b; Moser et al. 1997).

The 12 species from the genus *Elattoma* known to date are considered mycetophagous, and some of them can transport spores of pathogenic fungi (Rahiminejad et al. 2011). These species form phoretic relationships with several bark beetles (Rahiminejad et al. 2011) but are considered as rare and infrequent (Moser et al. 1989b); Hofstetter et al. 2013). This observation aligns with the results obtained for *Ips typographus* beetles but not for *I. duplicatus*, where specimens of the genus *Elattoma* were the most abundant, being, in fact, the only phoretic mite species that exhibited this preference. The only species from the genus *Elattoma* that forms phoretic relationships more with *I. duplicatus* than with *I. typographus* is *Elattoma crossi* (Khaustov et al. 2018). This species, which has been identified in the Siberian taiga (the native range of *I. duplicatus* beetles), may have been introduced to Romania with the migration of the beetles towards Southeast Europe. However, since the species could not be accurately identified, this remains only a hypothesis.

### Community diversity and structure

The species of phoretic mites and their abundance on the host varied between the native bark beetle species and the invasive species. Diversity indicates that the phoretic mite community of *I. duplicatus* is more homogeneous and uniform than the bark beetle community of *I. typographus*. Although the phoretic mite population of *I. typographus* was richer in species, the importance and weight of the dominant species, *D. quadrisetus*, was very high. This aspect is especially observable in the values obtained for the Berger-Parker index, which expresses the proportional importance of the most abundant species in a community (Berger and Parker 1970). The results of the PERMANOVA analysis show that the two communities differ significantly from each other. Similar results were obtained by Vissa et al. (2019), who analyzed the phoretic mite communities of three species of pine bark beetles in Portugal. These results further reinforce the proposed hypothesis that the phoretic mite communities of different species of bark beetles differ in terms of abundance and species structure. Although phoretic mite species, in general, exhibit a generalist behavior regarding host selection and are rather specific to certain habitats (Pfammatter et al. 2016), most species identified in this study recorded a higher number on *I. typographus* beetles compared to *I. duplicatus* beetles. It is possible that some species of phoretic mites, at the local level, may exhibit host specificity when selecting a host for transportation to a new subcortical microhabitat (Lindquist 1970), even though globally they are associated with a wide range of bark beetles. This hypothesis is supported by the findings of Knee et al. (2013), where, out of 29 analyzed species of bark beetles, approximately 70% of the identified phoretic mite species were associated with only one or two bark beetles. Factors such as the phenology, behavior of the bark beetle, or the microhabitat created by the host in its galleries may play a significant role in host selection (Knee et al. 2013).

Also, it is also important to note that, in areas where the two bark beetle species coexist, *I. duplicatus* often plays a secondary role, frequently accompanying *I. typographus* during the colonization of host trees (Duduman et al. 2013; Grodzki 2012; Wermelinger 2004). Consequently, phoretic mite species may preferentially choose the primary bark beetle species for transport over secondary species. This hypothesis is supported by findings from other studies, which have reported a higher number of phoretic mites on primary bark beetle species compared to secondary ones (Poliță et al. 2016; Vissa et al. 2019). Additionally, the fact that *I. duplicatus* is a relatively new species in the respective area cannot be overlooked, which may have limited its ability to establish strong relationships with all the mite species identified in this study. Another factor that may have influenced the choice of vectors for transporting phoretic mites is the size of the two species of bark beetles, with *I. duplicatus* being considerably smaller than *I. typographus* (Olenici et al. 2009), thus the surface area for attachment being reduced, particularly disadvantaging species that do not have specialized organs for attachment to the phoront or those that are larger, such as *P. austriaca* (Moser et al. 1989a).

### Localization of phoretic mites on the bodies of bark beetles

Phoretic mites typically attach to bark beetles using specialized structures such as the anal pedicel, chelicerae, or modified mouthparts (Bartlow and Agosta 2021). The distribution of phoretic mites is not random, certain areas of the host’s body are chosen based on the phoretic species (Houck and O’Connor 1991), as well as the possibility of being removed from the host’s body (Cejka and Holusa 2014). Studies have consistently shown that the area under the elytra is the most frequent attachment body part, followed by the thorax and abdomen (Cilbircioğlu et al. 2021; Gwiazdowicz et al. 2015; Manu et al. 2017; Moser and Bogenschuütz 1984; Moser et al. 1989a; Paraschiv et al. 2018; Paraschiv and Isaia 2020; Poliță et al. 2016), a pattern confirmed in this study for both *I. typographus* and *I. duplicatus*. This preference is primarily due to the high number of *D. quadrisetus* individuals, which predominantly occupy this area (Khaustov et al. 2018).

Other species exhibit distinct preferences, with *T. polytricha* and *U. ipidis* attaching to the thorax or abdomen using the anal pedicel, although intra and interspecific competition can cause them to occupy alternative sites (Paraschiv and Isaia 2020). In this study, *U. ipidis* was commonly found on the thorax and abdomen in *I. duplicatus*, and on the first pair of legs in *I. typographus*, while *T. polytricha* showed a clear preference for the elytral declivity region on both host species. *H. piceae*, *P. leontonychus*, and *P. fiseri* showed no specific attachment site preference on the host beetles’ bodies, a pattern also observed in other studies (Khaustov et al. 2018; Pernek et al. 2008). An interesting aspect of *Histiostoma piceae* is its documented hyperphoretic behavior with *U. ipidis* mites (Khaustov et al. 2016), while in this study, a similar behavior was observed with individuals of *D. quadrisetus* species.

*Elattoma* sp. did not exhibit a clear pattern of attachment on the body of *I. typographus* but were mostly found between the first and second leg pairs on the thorax and abdomen in *I. duplicatus*, aligning with Khaustov et al. (2018). The size of *P. austriaca* individuals, which makes it difficult to attach to a phoront, could explain why most specimens of this species were found under the elytra, a behavior also reported in other studies of species in this genus (Cilbircioğlu et al. 2021; Pernek et al. 2012).

## Conclusions

In this study, the populations of phoretic mites associated with two species of bark beetles were analyzed, the native species *I. typographus* and the invasive species *I. duplicatus*. The comparative analysis between the phoretic mite populations of these two bark beetle species highlighted considerable differences in terms of the rate of phoresy, the dynamics of the phoresy rate, as well as the structure and abundance of the two communities. The distribution of mites on the host bodies varied depending on the mite species and the abundance of mites on the hosts. Among the nine species of phoretic mites identified in this study, three are reported for the first time in Romania: *D. disetus*, *Elattoma* sp., and *P. leontonychus*. Although both the phoresy rate and its dynamics varied between the two hosts throughout the sampling period, the maximum phoresy for both bark beetle species was reached at the beginning of the flight of the hibernating generation, indicating this as the most important moment for the dissemination of mites into new habitats. The most abundant species on the bodies of *I. typographus* beetles was *D. quadrisetus*, which accounted for over half of the entire population. In the case of *Ips duplicatus* bark beetles, the population was dominated by specimens of *D. quadrisetus* and *Elattoma* sp., with the latter being the only species exhibiting this behavior in host selection.

## Data Availability

All original contributions presented in this study are included in the article and/or supplementary material. Additional supporting data can be obtained from the corresponding author upon request.
